# *I Move*: systematic development of a web-based computer tailored physical activity intervention, based on motivational interviewing and self-determination theory

**DOI:** 10.1186/1471-2458-14-212

**Published:** 2014-02-28

**Authors:** Stijn AH Friederichs, Anke Oenema, Catherine Bolman, Janneke Guyaux, Hilde M van Keulen, Lilian Lechner

**Affiliations:** 1 Faculty of Psychology and Educational Sciences, Open University of the Netherlands, P.O. box 2960, Heerlen, DL 6401, The Netherlands; 2 Department of Health Promotion, Maastricht University, P.O. Box 616, Maastricht, MD 6200, The Netherlands; 3 TNO, Expertise Center Life Style, P.O. Box 2215, Leiden, CE 2301, The Netherlands

## Abstract

**Background:**

This article describes the systematic development of the *I Move* intervention: a web-based computer tailored physical activity promotion intervention, aimed at increasing and maintaining physical activity among adults. This intervention is based on the theoretical insights and practical applications of self-determination theory and motivational interviewing.

**Methods/design:**

Since developing interventions in a systemically planned way increases the likelihood of effectiveness, we used the Intervention Mapping protocol to develop the I Move intervention. In this article, we first describe how we proceeded through each of the six steps of the Intervention Mapping protocol. After that, we describe the content of the *I Move* intervention and elaborate on the planned randomized controlled trial.

**Discussion:**

By integrating self-determination theory and motivational interviewing in web-based computer tailoring, the *I Move* intervention introduces a more participant-centered approach than traditional tailored interventions. Adopting this approach might enhance computer tailored physical activity interventions both in terms of intervention effectiveness and user appreciation. We will evaluate this in an randomized controlled trial, by comparing the *I Move* intervention to a more traditional web-based computer tailored intervention.

**Trial registration:**

NTR4129

## Background

Physical inactivity is the fourth leading cause of death worldwide and it increases the risk of many diseases such as coronary heart disease, type 2 diabetes, and breast and colon cancer [1]. Because of the beneficial effects of physical activity (PA), international guidelines state that for enhanced health adults should accumulate 30 minutes or more of moderate intensity PA on at least five days per week [2]. Unfortunately, large parts of the world population do not meet these guidelines and many individuals live a sedentary life [3]. This also applies to the Netherlands since in 2011 almost half of the Dutch adults were insufficiently active [4]. As a consequence, promoting changes in PA behavior is of great importance to public health [5]. There is a clear need for innovative PA interventions that are both efficacious and able the reach the broad population at-risk due to physical inactivity [6-8].

This article describes the development of a new and promising intervention approach to promote PA, by combining the technology of web-based computer tailoring (CT) with the theoretical insights and practical applications from self-determination theory (SDT) and motivational interviewing (MI). First, we provide some background information on CT, SDT and MI.

### Computer tailoring

Research indicates that PA interventions using CT are more successful than interventions in which generic information is provided [9-12]. In computer tailored interventions, the content of the provided materials is based on characteristics specific to each individual [13]. In the last decade, more and more computer tailored interventions are web-based, instead of print-delivered, which allows for more interactivity, higher accessibility and lower costs [11,14,15]. The findings of a recent meta-review suggest that internet-delivered PA interventions are useful in producing small but significant increases in PA [16].

Currently, the majority of web-based computer-tailored PA interventions have been grounded in theories such as social cognitive theory (SCT), the trans theoretical model (TTM) and the theory of planned behavior (TPB) [10,16,17]. Consequently, existing interventions mostly focus on theoretical constructs such as stages of change, modeling, attitude and self-efficacy. Current research on PA uptake and maintenance, however, increasingly shows the importance of another theoretical construct: autonomous motivation [18-21]. More specifically, increasing evidence shows that greater autonomous motivation predicts higher PA frequency and maintenance [22]. While autonomous motivation does not play an explicit role in SCT, TTM or TPB, it is a key construct in self-determination theory (SDT) and in motivational interviewing (MI) [23,24].

### Self-determination theory & motivational interviewing

SDT is a broad-based theory of human motivation [24-26]. Central to the theory is the distinction between autonomous and controlled motivation. Autonomous motivation includes intrinsic motivation (when someone is motivated to perform a certain behavior by enjoyment or satisfaction) and identified regulation (when someone is motivated by the pursuit of personally-valued outcomes). Controlled motivation consists of introjected regulation (when someone is motivated by the avoidance of negative emotions such as guilt) and external regulation (when someone is motivated by the prospect of rewards or the avoidance of punishments). Autonomous and controlled motivation are hypothesized both to strengthen and direct behavior, but to lead to very different outcomes, with autonomous motivation leading to greater commitment and long-term maintenance of behavior [24-27]. According to SDT, autonomous motivation is more likely to arise in an individual when the social context supports the basic psychological needs for autonomy (the need to engage in behavior with a sense of choice), competence (the need to feel competent and confident) and relatedness (the need to feel connected to and understood by others) [24-26].

Motivational interviewing (MI) is defined as “a collaborative conversation style for strengthening a person’s own motivation and commitment to change” [23]. The core or “spirit” of MI encompasses principles of partnership, acceptance, compassion and evocation. Along with these principles, four overlapping processes are discerned in MI: engaging, focusing, evoking and planning. In order to carry out these principles and processes, the practice of MI implicates applying specific communication skills: asking open questions, affirming, reflective listening, summarizing and informing/advising [23]. Although SDT and MI were developed independently of one another, several scholars claim that SDT can offer a theoretical framework for deepening our understanding of the efficacy of MI [28-30]. More precisely, it is argued that the specific strategies in MI fulfill the clients’ basic psychological needs for competence (e.g. by using strategies to explore and build confidence), autonomy (e.g. by allowing clients to discover their own reasons for change), and relatedness (e.g. by being compassionate) [29].

### SDT & MI in physical activity promotion

As SDT has received substantial empirical support in the context of PA, researchers have begun to implement PA promotion interventions grounded in SDT [31]. In these interventions, strategies are implemented that are aimed at supporting the three basic psychological needs. By doing so, these interventions try to help the individual to develop more autonomous forms of motivation towards adoption and maintenance of PA [31]. The first RCT’s to test SDT interventions aimed at PA all show intervention effects albeit of different formats [31].

As discussed above, it is assumed that MI can be used to fulfill the individuals’ basic psychological needs [29]. MI has become increasingly popular among PA counselors [32]. Evidence illustrates that motivational interviewing can be successful in getting individuals to increase their PA [33].

### SDT & MI in web-based CT

There are several arguments for integrating SDT and MI in web-based CT. Firstly, previous research by Resnicow and colleagues showed that tailoring on SDT and MI principles in *print-based* CT can be successful [34]. Web-based CT, however, offers more opportunity to simulate an interactive, collaborative conversation when compared to print-based CT [14,35]. In web-based CT information can be tailored to answers immediately after they have been given. As a consequence, interactive features that contribute to the sense of collaboration can be more easily included in web-based CT. Secondly, delivering MI and SDT-based interventions in a traditional way (face-to face by a clinician), is expensive and difficult to scale up. Compared to face-to-face counseling, web-based interventions in which SDT and MI techniques are embedded, better enable the dissemination of these client-centered counseling techniques and may be more cost-effective [6]. Thirdly, compared to traditional web-based CT, web-based CT grounded in SDT and MI may enhance intervention efficacy because of its focus on promoting autonomous motivation.

In this article, we aim to provide insight into the development and content of the *I Move* intervention, a web-based computer tailored physical activity intervention based on the theoretical insights of SDT and practical applications of MI.

## Methods/Design

*I Move* (*Ik Beweeg* in Dutch) is a web-based computer tailored PA promotion intervention, aimed at increasing and maintaining PA among adults. This intervention is based on the theoretical insights and practical applications of SDT and MI.

Since developing interventions in a systemically planned way increases the likelihood of effectiveness [36], we used the Intervention Mapping (IM) protocol [37] to develop the *I Move* intervention. The IM protocol consists of six succeeding steps that can be used as a guide for theory and evidence based decision making during intervention development, implementation and evaluation [37]. We first provide an overview of how we proceeded through each of these six steps. After that, we will elaborate further on the development of the *I Move* intervention, and on the evaluation plan.

The **first step** of the IM protocol concerns an assessment of the health problem and its related behavior [37]. As discussed in the introduction, physical inactivity has major health effects such as increased risk of many adverse health conditions [1]. Therefore, this study focuses on physical inactivity in the general population. The desired behavior for adults is to increase their PA level (and to maintain this increased PA level).

The purpose of the **second step** of IM is to formulate change objectives [37]. Change objectives are composed of performance objectives (i.e. sub-behaviors within the desired behavior) and determinants of behavior change. We first formulated performance objectives such as “deciding to become more physically active” and “developing a plan of how to become more physically active”. Since the most important theoretical framework in this study is SDT, the most relevant determinants that need to be targeted are autonomy, competence and relatedness [24-26]. Next, we defined several change objectives. Change objectives specify what skills individuals have to learn in order to accomplish the performance objectives and are created by combining the performance objectives and the determinants (autonomy, competence and relatedness). Examples of the change objectives for the *I Move* intervention are: “Adults can express why becoming more physically active is important for them personally”, “Adults are able to make plans that suit well to their personal preferences” and “Adults are able to make plans about which they feel confident”.

During the **third step** of IM, theoretical methods and practical applications for behavior change are selected [37]. A theoretical method is a general process that is intended to change behavioral determinants. A practical application concerns the way of putting such a theoretical method into practice in the actual intervention. As discussed in the introduction, it is assumed that the specific strategies in MI can help the individual towards behavior change by supporting the need basic psychological needs for autonomy, competence and relatedness. Therefore, we chose MI as the main theoretical method in this study. As the practical application we translated the skills, processes and spirit of MI into web-based computer tailoring. We will elaborate on this process in the subsequent section.

The **fourth step** of IM is about the development of the actual intervention [37]. During this step, the intervention is also pretested. In the section below we will elaborate on the specific content of the *I Move* intervention and on the pretest.

In the **fifth step** of IM, a plan for program adoption and implementation is developed [37]. Since this study is one of the first to integrate MI in web-based CT, however, we chose to focus on adoption and implementation in context of the evaluation study. The intervention was set up in such a way that minimal human action is required for implementation. The intervention is available via a website. Individuals who want to use the intervention can go to the website to sign in. Participants in the study automatically receive invitations by email to visit the study website, every time a new intervention session is available. In order to increase the likelihood of repeated program use (e.g. completing all intervention sessions) we interviewed potential users from the target group about their preferences and needs regarding the content and appearance of the intervention and the recruitment materials for the evaluation study. In order to further optimize recruitment for the study, we prepared a detailed recruitment protocol.

During the **sixth step** of IM a plan is made for the process and effect evaluation of the intervention. In order to evaluate the efficacy of the *I Move* intervention, we will conduct a randomized controlled trial (RCT). We will elaborate in more detail on this step in the section below.

### Translation of SDT and MI into web-based CT

This section presents an overview of how the theoretical insights and practical techniques of SDT and MI were translated into web-based CT. More specifically, we describe how the skills, processes and spirit of MI are implemented in the *I Move* intervention. Obviously, application of these elements in an automated, web-based platform differs from application in a real life counseling setting. Indeed, a face-to-face setting allows expression of genuine empathy and responding to very subtle expressions of motivation, which is less feasible in an automated platform. On the other hand, web-based CT allows for high interactivity and very specific feedback messages which advances similarity to face-to-face counseling.

### Translation of MI skills into web-based CT

The practice of MI involves the use of several core communication skills that are needed throughout the whole counseling process. These are asking open questions, reflective listening, affirming, summarizing and informing/advising. Here we describe how we translated these counseling skills into web-based CT.

### MI skills: asking open questions and reflective listening

Two of the core skills of MI are asking open questions and reflective listening [23]. Asking open questions is important in MI, since open questions offer more space for the participants to come up with their own answers, insights and reasons for change [23,38]. SDT also underlines the importance of asking open questions, since it offers more opportunity for autonomy support when compared to a limited set of response options [38].

Reflective listening, derived from Carl Rogers’ client-centered therapy, involves understanding the client’s idea, thoughts and feelings, and to relay this understanding back to the client. In MI, this is also referred to as complex – or skillful – reflections [39,40]. These reflections typically add substantial meaning or emphasis to what the client has said; they serve the purpose of conveying a deeper or more complex picture [39,40].

An important challenge during the development of the *I Move* intervention was to implement reflective listening in web-based CT. Unlike a human counselor, a web-based intervention is not able to really interpret answers to open questions. We tried to assemble skillful reflections by using a structured approach, with multiple choice questions. We developed a unique feedback message for each combination of multiple choice answers. In the feedback message we tried to add meaning to the combination of answers, in order to add a dimension of understanding and ‘skillfulness’ to the message.

**Figure 1:**
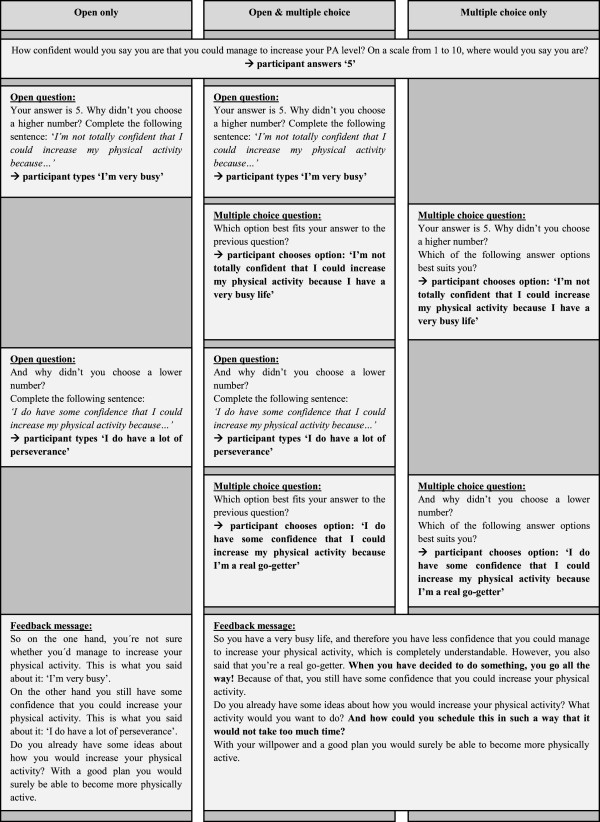
Three tailoring methods.

Open questions, however, are also an important core skill of MI, and these are not used in the above example. In order to find out how to combine skillful reflections and open questions in web-based CT, we conducted a pilot study [41]. In this study, we compared three interventions based on MI and SDT principles: (i) exclusively open questions (without skillful reflections), (ii) exclusively multiple choice questions (with skillful reflections) and (iii) including both question types (with skillful reflections). Figure 1 shows (simplified) examples of the tailoring mechanisms in the three intervention conditions from this study. The intervention with both open and multiple choice questions yielded the best results. Individuals who received this intervention increased their commitment towards regular PA, and they were most positive about the intervention. Therefore, the approach with both open- and multiple choice questions was used in the *I Move* intervention.

### MI skills: affirming

In MI, the counselor respects the client as being capable of growth and change. By using affirmative reflections, the counselor recognizes and comments on the client’s particular strengths and efforts [23]. Affirming is also relevant from the perspective of SDT since it nurtures the client’s need for competence and relatedness [29]. We implemented affirming in the *I Move* intervention by using an empathetic style in the feedback messages. For example, if a participant failed to become more active, the feedback rewards the participant for trying, and stresses that a failed try does not mean that he or she is incapable.

### MI skills: summarizing

Summaries are reflections that pull together several things that the client just has told. These help the client to reflect on the various experiences that he or she expressed [23]. In addition, summarizing supports the client’s basic psychological need for autonomy by hearing his or her own thoughts and ideas back from the counselor; and competence by structuring the conversation [29]. During the *I Move* intervention participants regularly receive messages that summarize the before going. For example, after discussing the importance of PA, the participant receives a feedback message that summarizes what he or she answered to the prior questions. One day after each of the four intervention sessions, the participants receive an email with a pdf attached, in which the matters discussed during the last session are summarized.

### MI skills: informing and advising

In MI, information exchange is viewed as a collaborative search to understand the client’s information needs [23]. First, the MI counselor clarifies information needs and gaps: he finds out what the client wants and needs. If an information need exists, the counselor asks permission to provide information. Information is presented in such a way that the counselor provided information to the client, without interpreting this information any further [23]. From the viewpoint of SDT, this approach of providing information supports the client’s basic psychological need for competence [29].

In the *I Move* intervention, participants are offered the possibility of receiving information through several short expert videos. In these videos, a doctor tells about the positive effects of regular physical activity in several domains such as physical health, mental health, physical appearance, and social life. Participants are given the option to choose for themselves which videos they want to see. This approach of providing information is congruent to the practice of MI (and to the principles of SDT), since the intervention assesses the individual need and desire for information, before providing it.

### Translation of MI processes into web-based CT

In the practice of MI four overlapping processes are discerned: engaging, focusing, evoking and planning. Here we show how we translated these processes into web-based CT.

### MI processes: engaging

In MI, engaging concerns involving the client in the counseling process and establishing a trusting and respectful helping relationship between client and counselor [23]. Similar to MI, SDT also acknowledges the importance of involving the client [29,38]. In the *I Move* intervention the participants are involved in the process in several ways. Firstly, at the start of the intervention, they receive information about the nature of the intervention. At the start of each intervention session, the participants receive an overview of the content of that session. It is stated that the participants are experts about themselves and that *I Move* can help them to get a clear view on their own preferences, needs and goals concerning PA.

As mentioned above, the process of engaging also involves establishing a social relationship between client and counselor. The importance of such a bond is underlined by SDT since it supports the basic psychological need for relatedness [38]. One possible method to facilitate the development of a such a social relationship between user and program is to use an anthropomorphic agent, i.e. a virtual coach that has a face and body [42-44]. Research on this topic demonstrated that users can successfully form a working alliance with a virtual coach [45,46]. Also, multiple studies on web-based interventions confirmed that the presence of a virtual coach can further improve intervention effectiveness [47-49].

**Figure 2:**
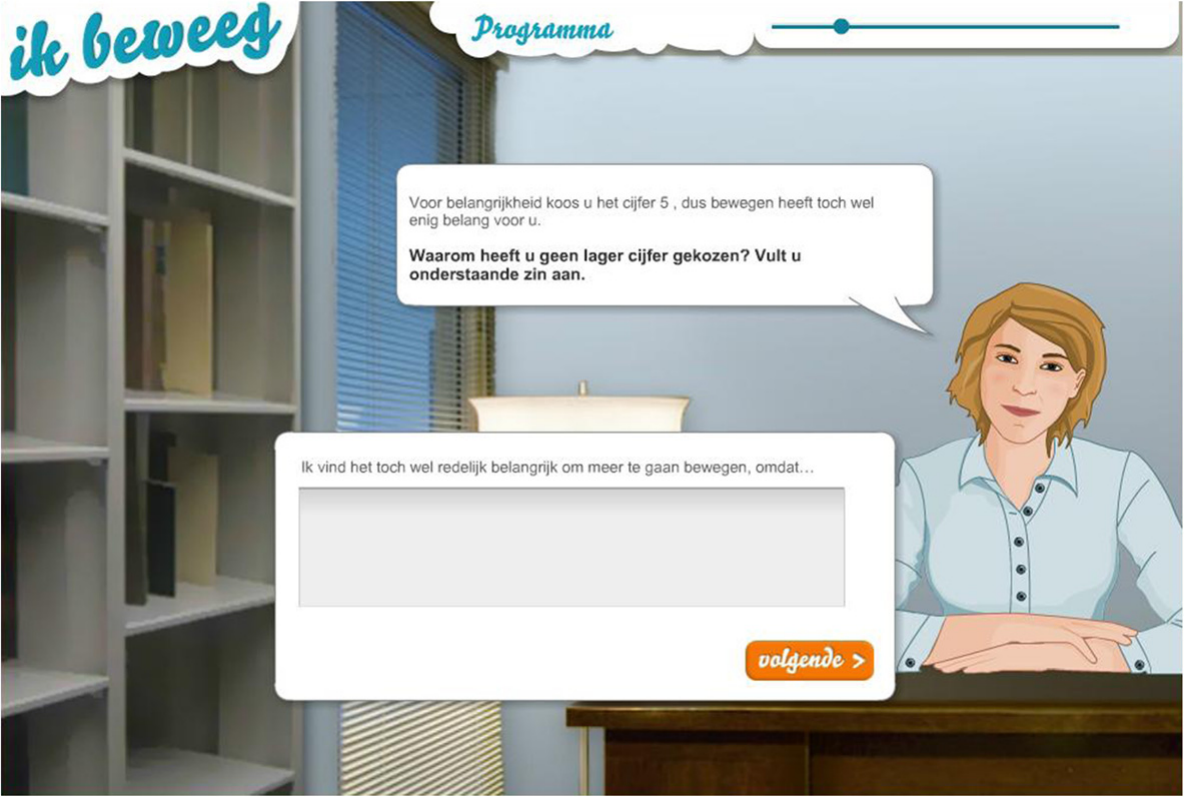
SDT/MI intervention with a virtual coach.

**Figure 3:**
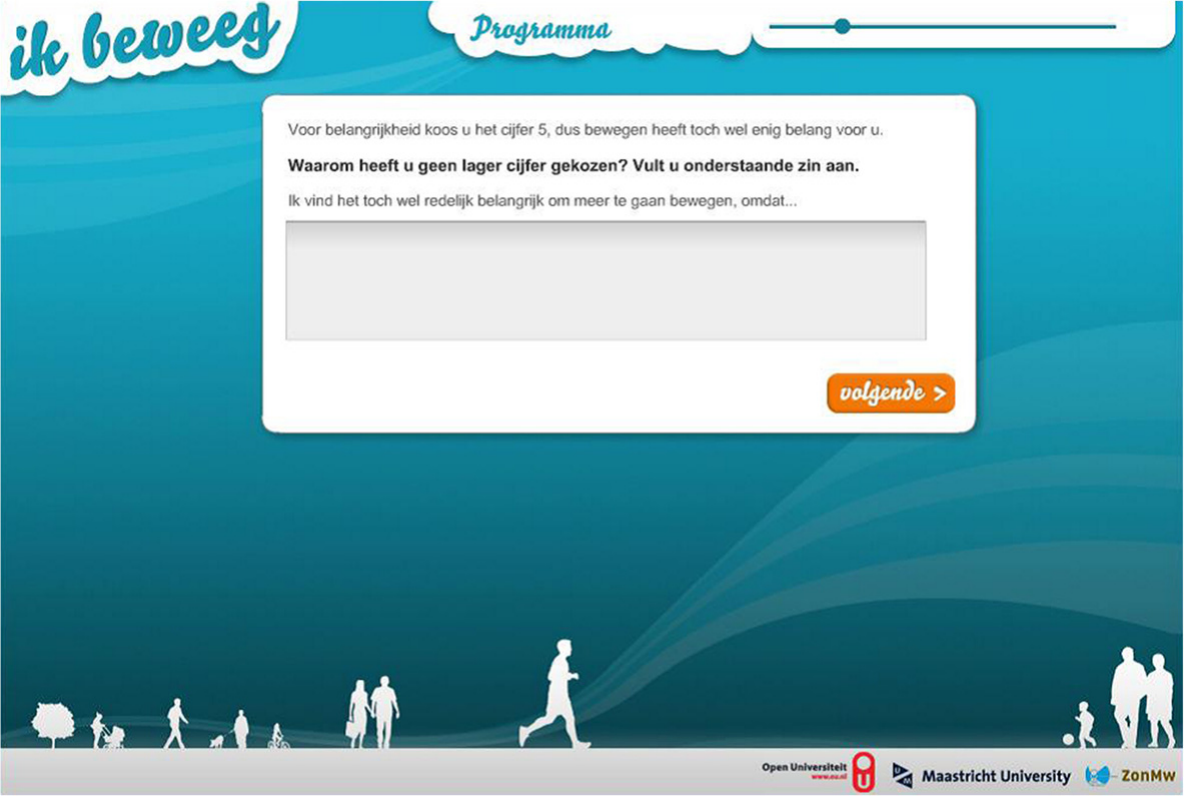
SDT/MI intervention without a virtual coach.

In order to find out whether inclusion of a virtual coach would be beneficial, we conducted a second pilot study [50]. In this study we compared three intervention conditions: a web-based SDT/MI intervention with a virtual coach, 2) a content identical, text-based intervention without a virtual coach and 3) a control condition receiving no information (see Figures 2 and 3 for an example of both interventions). The results showed that the presence of a virtual coach did not result in significantly more PA than the other condition, nor did it result in more appreciation for the intervention [50].

The virtual coach in this study was designed in a cartoonish way, without the use of voice. This design could have led to less-than-optimal perceived realism of the coach [51]. Therefore, a more realistically shaped coach – which is also perceived as being more realistic – might be more successful in web-based SDT/MI interventions. In practice, such an endeavor can be achieved by creating a video-coach: a series of video messages, in which a real human coach speaks to the participant. Previous research shows that this type of interactive video-counseling holds promise for public health interventions [52-54].

**Figure 4:**
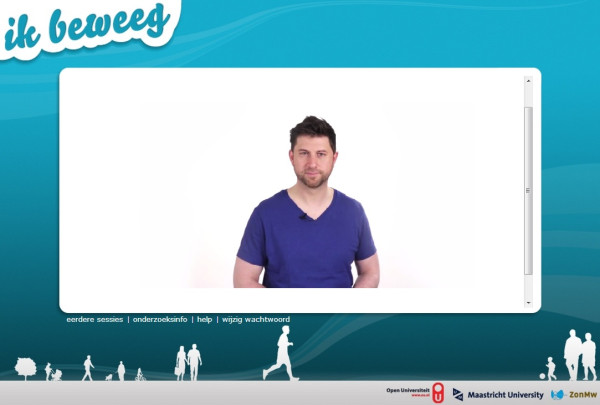
**The video-coach.** (The individual shown agreed with publication of this image).

Therefore, in the *I Move* intervention we chose to include a *video-coach*, a series of video messages in which a real human coach speaks to the participant (see Figure 4 for an example). Each of the four sessions starts with a short video, in which the coach welcomes the participant and briefly introduces new questions and exercises (for example about motivation or confidence).

Furthermore, on several moments throughout the intervention sessions, participants can watch short videos with narratives of four allegedly “former participants of *I Move*” [55]. These videos appear throughout the whole intervention, and depict four chronological storylines of those four former participants. In these videos, the former participants tell for example about what they think is important with regard to being physically active, and about how they have managed to gain more confidence to be active. The four former participants differ in age, gender (one younger woman, one younger man, one older woman and one older man), and experience in PA (varying from active sports participation to experience with recreational activities). These videos are always preceded by a video in which the coach gives a brief introduction of the videos. Participants can choose whether they want to watch the videos, and which one (s) they want to watch. These narratives were implemented because they provide the opportunity for the participants to feel connected with others who go through similar experiences as themselves, which may enhance relatedness [38].

### MI processes: focusing

In MI, focusing is a collaborative process of finding topics for conversation and consultation [23]. Since the *I Move* intervention is one of the first efforts to integrate MI in web-based CT, however, we have limited the scope to PA. Before entering the intervention, the participants are told that the intervention is about PA. Within the context of PA, participants are encouraged to come up with their own themes and ideas.

### MI processes: evoking

In the evoking process of MI, it is intended to elicit the client’s own motivation for change by prompting self-determined motivational statements or change talk [23]. Furthermore, it is intended to help the client increase his/her perceived confidence. These goals are in line with SDT's emphasis on autonomy and competence [29]. In the *I Move* intervention, evocation is pursued through several methods, derived from MI [23,56]. The most important of these methods are described below. For each of these methods, it is described how it is has been implemented in the intervention (i.e. how the several counseling strategies have been translated into automatized text versions).

### Importance ruler

In this method, the participants are asked to rate their perceived importance on a scale [56]. In the intervention, the method starts with this question: “How important would you say it is for you to become more physically active? On a scale from 1 to 10, where would you say you are?”. If a mediocre score (4–7) is chosen, these questions follow: 1) It looks like becoming more physically active is not totally unimportant to you, but neither is it totally important, why did you not choose a higher number?’ and 2) ‘Why did you not choose a lower number?’. These two questions are asked to identify barriers and to prompt self-determined motivational statements. If a high score (8–10) is chosen, this question follows: ‘Why is becoming more physically active so important to you?’. This question is asked to prompt self-determined motivational statements. If a low score (1–3) is chosen, this questions follows: ‘It looks like becoming more physically active is not on top of your list. Maybe you still have reasons why becoming more physically active could be important to you though. Why could becoming more physically active be important to you?’ This question is asked to prompt self-determined motivational statements. After answering the follow up questions, the participants receive a reflective feedback message which also contains a summary of the participant’s own answers. For the participants with a mediocre score, this feedback message mainly elaborates the discrepancy between the barriers and self-determined motivation. For the participants with a high score, the feedback message emphasizes the participant’s strong motivation, including the most important reason for this (your most important reason for this was: ‘being physically active is good for my health’). The feedback messages for the participants with the low score elaborates on the discrepancy between the low score for importance, and the motivational statement made in the follow up question.

For the importance ruler section we developed 80 different feedback messages to increase fit to individual’s answers. This is an example of such a feedback message: “*On the one hand you think it is not that important to become more physically active because you have a very busy life. On the other hand you do think it is important to become more active because physical activity helps you to relax. You also say: “I always feel very relaxed after exercising.” Nowadays many people have a very busy life, full of activities like working, social contacts, housekeeping, watching TV and on and on. For many people, this modern life is very busy and they need to make choices between their activities. This is a very common and very understandable situation. Many people find it convenient to deal with this situation by assessing what their current activity schedule is like on a typical day in their lives. As the next step, they decide whether they spend enough time doing things they really think are important. Maybe this could be an interesting idea for you, too?”*

### Value clarification

This technique is often used in motivational interviewing. In the intervention, the participant is first asked “What would you say are your two most important values in life?”. After choosing two values, the participant is asked how becoming more physically active may relate to these important values. The respondent is asked to fill in his answers. At the end of this assignment the participant gets an overview of these answers and he or she is asked whether it might be interesting to contemplate a bit further on this subject.

### Looking forward

The participant is asked to imagine that he or she would become more physically active and that he or she would maintain this new level of PA for a period of five years. As the next step, the participant is asked to imagine what possible beneficial effects such an increase in PA would entail for him or her personally. How would he or she feel, and would this consideration have influence on how he or she feels about being more physically active.

### Confidence ruler

This technique is comparable to the importance ruler. In our intervention, it starts with this question: “How confident would you say you are to become more physically active? On a scale from 1 to 10, where would you say you are?”. If a mediocre score (4–7) is chosen, these questions follow: 1) It looks like you have some confidence, but you are not totally confident, why did you not choose a higher number?’ and 2) ‘Why did you not choose a lower number?’. These two questions are asked to identify barriers and to prompt facilitators or personal strengths. If a high score (8–10) is chosen, this questions follows: ‘why are you so confident that you could become more physically active?’. This question is asked to prompt facilitators or personal strengths. If a low score (1–3) is chosen, this questions follows: ‘It looks like you are not very confident. What would help you to accumulate more confidence?’. This question is asked to stimulate the participant to think about ways to increase their confidence. After answering the follow up questions, the participants receive a reflective feedback message. For the participants with a mediocre score, this feedback message mainly elaborates the discrepancy between the barriers and facilitators. For the participants with a high score, the feedback message emphasizes the participant’s strong confidence and personal strengths. The feedback messages for the participants with the low score mainly elaborate on how the participant could become more confident.

For the confidence ruler section we developed 87 unique feedback message, so the feedback fits optimally to each individual’s answers. This is an example of such a feedback message: *“Because of an unsuccessful attempt to become more active in the past, you are not totally confident that you could manage to succeed in becoming more active. However, you also say that you really want to become more active. Because of that, you do have some confidence that you could succeed. You also say: “Once I want something, I’m unstoppable.” Really wanting something is very important in order to achieve it! Also, if you were unsuccessful in achieving something once, that doesn’t mean that you are not able to do it, it is just a learning experience! Do you already have an idea of how you would approach becoming more active? What went wrong last time? What can you do differently this time, to avoid failure? With a good plan, you can certainly succeed in becoming more active, especially if you really want to!”*

### Looking back

This method is intended to increase the participant’s perceived confidence by asking about past successful experiences. The following is asked in the intervention: ‘I would like to ask you to think back of a moment in time when ultimately you succeeded in doing something very difficult. What did you have to do? How did you ultimately succeed? How did you feel when you had succeeded? After typing these answers, an overview of these answers was presented and the following question was asked: ‘Could looking back to this experience help you in becoming more confident that you also could succeed in becoming more physically active?’

### MI processes: planning

In MI, the planning process is seen as the bridge to change: by developing a specific change plan people are more likely to follow through [23]. Indeed, evidence shows that behavior change is more likely to occur when people translate their intentions into specific plans [57-59]. In the *I Move* intervention, participants are offered the opportunity to create a specific action plan by proceeding through a structured planning module. In this module, they are asked to formulate their most important motivation for becoming more physically active, the activity or activities and location (s) of their choice, on how many days a week they want to execute the activity and how long they want to spend on the activity on such a day. If desired, the participants can also indicate with whom they want to perform the activity and what preparations they need to undertake before being able to implement their plan.

Participants who choose to not make an action plan in their first *I Move* session are given the option to create such a plan in the following session. When a participant has made an action plan, the execution of this plan will be evaluated in the subsequent session. By doing so, the participants are stimulated to reassess the feasibility of their self-made plans. They are encouraged to identify factors that hindered or supported them in realizing their plan. Furthermore, participants are able to adjust their plan to their needs and wishes in each follow up session.

Participants are also given the option to create coping plans. Coping planning refers to anticipating on barriers, such as difficult situations or unwanted distractions that might hinder performing the planned behavior. It includes analyzing possible barriers and detailed planning on how to overcome these barriers [57-59]. Such coping plans consist of an *if-component* which represents the difficult situation and a *then-component* which represents the planned strategy of coping with this situation [58]. First, the participant is asked to identify two difficult situations, in which it would be challenging to enact their action plan. Then, he or she is asked to come up with solutions to cope with these situations. Finally, the participant gets an overview of his/her difficult situations and coping solutions.

### Translation of the MI spirit into web-based CT

In MI, the spirit refers to the underlying perspective, the set of heart and mind with which one enters into the practice of MI. The spirit includes applying elements of partnership, acceptance, compassion and evocation [23]. Here we describe how we developed the intervention as congruent as possible to these four principles.

### MI spirit: partnership

The element of partnership refers to the vision of MI, that the counseling process is conducted together ‘with’ the client, rather than ‘to’ the client [23]. Therefore, in MI the counselor is explorative and interested, rather than coercive and persuasive. This approach supports the client’s basic psychological need for relatedness [29]. In the *I Move* intervention the participants are constantly asked to give their opinion, or to reflect on a statement they made earlier. In this manner the *I Move* intervention uses the participant’s input as the starting point for each session, to build up a collaborative conversation.

### MI spirit: acceptance & compassion

The practice of MI involves an attitude of profound acceptance for the client, and a priority for the client’s needs [23]. As mentioned above, the *I Move* intervention uses the participant’s input as the starting point to build up a collaborative conversation. We also put a lot of effort into developing highly specific feedback messages, that suit well to the client’s needs and wishes. Furthermore, we wrote all feedback messages using an empathetic style, without using coercion or blame.

### MI spirit: evocation

The element of evocation refers to the premise of MI, that the clients already have much within them of what is needed and the counselor’s task is to help finding it [23]. As discussed above, the *I Move* intervention contains many elements that are intended to evoke the participant’s own motivation (i.e. value clarification) and confidence (i.e. confidence ruler).

### Content of the four intervention sessions

**Figure 5:**
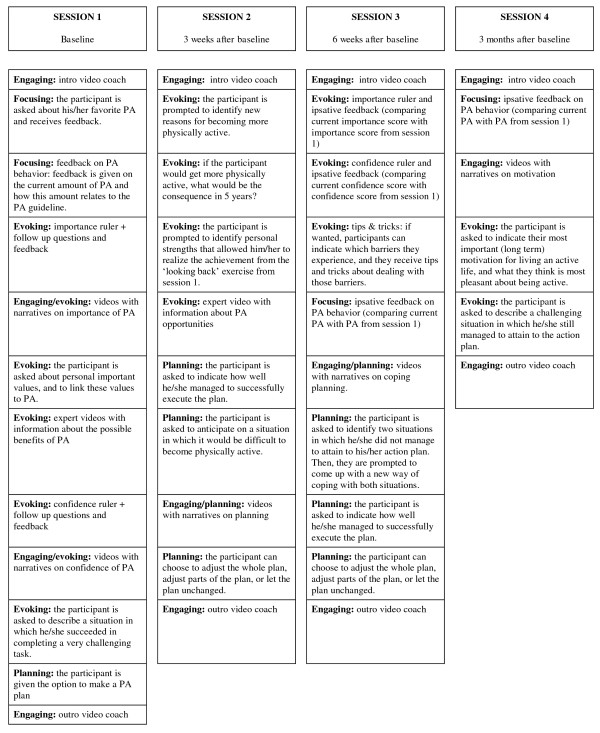
Content of the intervention sessions.

The *I Move* intervention consists of four sessions (see Figure 5). Before session 1, 3 and 4 the PA of the participants is assessed using a validated web-based PA questionnaire [60]. The results of this questionnaire are used to compose the feedback on the participant’s PA level. Of the four interactive sessions, session 1 is the most extensive. This session starts with an introductory part, and after that several topics are discussed such as the participant’s current PA (derived from the results from the questionnaire), how important he or she finds it to become more physically active, and how confident he or she is with regards to becoming more physically active. Participants can choose whether or not they want to make an action plan to become more active. In a previous pilot study, 85-90< chose to make such a plan [41]. Three weeks and six weeks after session 1, participants receive an invitation email for session 2 and 3, respectively. As can be seen in Figure 5, session 2 and 3 mainly further elaborate on importance and confidence of becoming more physically active. Participants are given the option to evaluate and adapt their plans, and formulate coping plans. Three months after session 1, participants can enter session 4. At the beginning of this session the participants choose which parts of the session they want to go through. They can choose to go through all parts (ipsative feedback on PA, long term motivation and confidence), but they also can decide to skip one or more of these parts.

### Pretest

Once the intervention was technically tested and there were no more bugs, a pretest was conducted among eight individuals from the target group. All individuals worked through the sessions at home, from their own computer or tablet. They were asked to fill in a printed questionnaire assessing their user experience during working through the sessions. Furthermore, five of the eight participants were observed and interviewed while they were working through the intervention. The pretest yielded several suggestions for improvement. Many of these comments were about the lay-out of the website. In addition, there were some comments concerning the PA assessment questions. Several pretesters experienced difficulty for they did not know how to correctly fill in the questions on hours and minutes spent on a physical activity per day. Based on these results, the intervention was improved and some questions were clarified through additional instructions. After this, the intervention was tested technically once more by four members of the research group.

### Evaluation design

**Figure 6:**
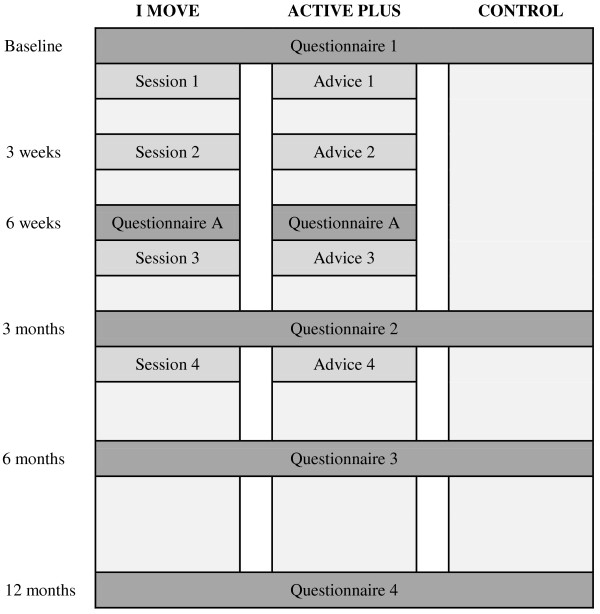
Evaluation design.

The *I Move* intervention will be evaluated on efficacy and appreciation in an RCT. Figure 6 shows the timeline of this study. In this RCT, three research groups will be compared:

1) I MOVE: This group receives the *I Move* intervention.

2) ACTIVE PLUS: This group receives a more traditional web-based computer tailored PA intervention. This intervention was derived from the *Active Plus* intervention [61,62]. The *Active Plus* intervention is a systematically developed theory- and evidence-based intervention which is predominantly based on traditional health behavior change theories such as TPB, SCT and TTM. The *Active Plus* intervention, which is aimed at PA increase and maintenance, consists of three times a tailored advice, and is designed for individuals over 50 years [61,62]. For the purpose of this study, the intervention was adapted in such a way that it is suitable for the general adult population. Since the original *Active Plus* interventions contains only three times a tailored advice, we added one extra tailored advice, in order to make it optimally comparable to the *I Move* intervention (which contains four sessions).

3) CONTROL: This group consists of a waiting list control-condition. The participants in this research condition gain access to the *I Move* intervention as soon as they finish the last evaluation measurement at one year.

By comparing these research conditions, it will be possible to evaluate whether the *I Move* intervention is more effective than no intervention, and whether it is more effective or better appreciated than the traditionally tailored intervention. Figure 6 shows an overview of the study. Measurements are taken at baseline and at 3, 6 and 12 months from baseline. All measurements are taken by web-based questionnaires via the study website. Participants from the intervention conditions are also asked to fill in a questionnaire at 6 weeks from baseline (‘Questionnaire A’ in Figure 6). This questionnaire contains questions that are needed in order to provide tailored feedback in session/advice 3 as well as items that assess process evaluation regarding session 1 and 2. Therefore, this questionnaire is not presented to participants from the control condition.

This study is approved by the Medical Ethics Committee of Atrium–Orbis–Zuyd and was registered with the Dutch Trial Register (NTR 4129). All eligible individuals who choose to participate in the study are asked to sign an online informed consent form.

### Participants

Participants are eligible for participation when they are between 18 and 70 years old, do not have a condition that seriously affects their ability to be physically active, did not participate in one of the *I Move* pilot studies, and when they are less physically active than 5 days per week 60 minutes per day. Participants will be recruited by advertisements in national newspapers, social media, and via an online panel. Based on a power calculation (ES = .25; power = .80), data of 600 participants are needed for this study. Based on a dropout rate of 40-70< [63,64] a minimum of 2000 respondents will need to enroll in the study.

After passing through the inclusion questions and giving informed consent, participants are randomized into one of the three research conditions and fill in the baseline questionnaire. In order to decrease attrition, 10 prizes of €50 will be raffled among those participants who have completed each questionnaire [65]. Among those participants who completed all questionnaires, two tablet computers are raffled.

### Measurements

The primary outcome for this study is PA behavior. Total weekly days of sufficient PA and minutes of moderate to vigorous PA are assessed using the validated self- administered Dutch Short Questionnaire to Assess Health Enhancing Physical Activity (SQUASH) [60]. Total weekly days of sufficient PA is measured by a single item: ‘How many days per week are you, in total, moderately physically active by undertaking, for example, brisk walking, cycling, chores, gardening, sports, or other physical activities for at least 30 minutes?’. Total weekly minutes of moderate to vigorous PA is computed by multiplying the frequency (how many days per week), and duration (how many hours and minutes per day) of leisure and transport walking, leisure and transport cycling, sports, gardening, household chores and odd jobs performed with moderate or vigorous intensity.

In addition to PA, several secondary measures are assessed. Motivational regulation towards PA is assessed using the Exercise Self-Regulation Questionnaire (SRQ-E). The SRQ-E contains subscales that represent external regulation, introjected regulation, identified regulation, and intrinsic motivation [66]. The Intrinsic Motivation Inventory (IMI) is used to assess the feelings that participants experience while being physically active [67]. The IMI encompasses the following subscales: interest/enjoyment, perceived competence, effort/importance, perceived choice, usefulness and pressure/tension. In addition, intention [68] and commitment [69] towards being physically active are assessed. As a possible moderator, individual preference for autonomy support is assessed using a single item, derived from a study by Resnicow et al. [34]: “In general, when it comes to physical activity I would rather an expert just tell me what I should do”.

The extent to which the intervention supports the basic psychological needs for competence, autonomy and relatedness are assessed by nine self-constructed items (three for each of the basic psychological needs). In addition, the participants are asked to what extent they perceived the content of the intervention as useful, understandable, believable, personally tailored and motivating. These items were derived from another study on web-based CT [70].

## Discussion

The aim of this article was to describe the systematic development of the *I Move* intervention: a web-based PA intervention based on SDT and MI. The *I Move* intervention consists of four automated web-based sessions. In these sessions, participants can proceed through several text-based questions, feedback messages and videos. The *I Move* intervention is developed in systematic way, based on theory and evidence, using the IM protocol [37]. Since developing interventions in a systemically planned way increases the likelihood of effectiveness, this approach represents an important strength of this intervention [36].

The *I Move* intervention is one of the first attempts to integrate MI and SDT in web-based CT. As discussed above, most of the current web-based computer tailored PA interventions have been grounded in SCT, TTM and TPB [16]. Although these interventions are more effective and are perceived more positively than interventions in which general information is provided, effect sizes are rather small [9-12,16]. By integrating SDT and MI in web-based CT, the *I Move* intervention introduces a more participant-centered approach. Adopting this approach could enhance intervention effectiveness and user appreciation. We will evaluate this by comparing the *I Move* intervention to a more traditional web-based computer tailored intervention. We will also assess whether the participant-centered approach as adopted by the *I Move* intervention is more beneficial for subgroups of individuals, for example those individuals who prefer an autonomy supportive style of communication [34].

In the *I Move* intervention several MI strategies have been implemented. In order to do so, we translated the skills, processes and spirit of MI into automatized text versions. Throughout this process, we used SDT as the underlying theoretical framework [29]. Nonetheless, there are differences between the *I Move* intervention and MI or SDT-based counseling in a face-to-face context. For example, compared to a web-based intervention, a real human counselor is better able to provide skillful reflections and to respond to very subtle expressions of motivation to change. By optimally using the possibilities of web-based CT, we tried to achieve a high degree of similarity to a face-to-face counseling situation. For example, by using a combination of open questions and multiple choice questions, we were able to provide very specific feedback messages while allowing the participants to come up with their own answers [25].

Another important difference between the I Move intervention and face-to-face counseling concerns the financial aspect. When compared to the costs of face to face counseling, a web-based delivery mode enables a higher reach since implementation costs are very limited [6]. Since intervention impact equals the product of efficacy and reach, this consideration should be taken into account when comparing web-based and face-to-face counseling interventions [7].

This article describes the content of *I Move*, and provided an example of how SDT and MI can be translated into web-based computer tailoring. The results of the RCT will provide more insight into the value of this novel intervention type. This knowledge can then be used for further development and optimization of web-based interventions, in the domain of PA and in other behavioral domains.

## Abbreviations

CT: Computer tailoring; IM: Intervention mapping; MI: Motivational interviewing; PA: Physical activity; RCT: Randomized controlled trial; SCT: Social cognitive theory; SDT: Self-determination theory; TPB: Theory of planned behavior; TTM: Trans theoretical model.

## Competing interests

The authors declare that they have no competing interests.

## Authors’ contributions

LL, AO, CB and HvK designed and wrote the original proposal. SF, AO, CB, JG, HvK and LL developed the intervention. SF significantly contributed to writing this article. AO, CB, JG, HvK and LL were involved in revising the manuscript critically. All authors read and approved the final manuscript.

## Pre-publication history

The pre-publication history for this paper can be accessed here:

http://www.biomedcentral.com/1471-2458/14/212/prepub
